# Biological properties of experimental dental alginate modified for self-disinfection using green nanotechnology

**DOI:** 10.1007/s00784-023-05277-8

**Published:** 2023-09-30

**Authors:** Lamia Singer, Sabina Karacic, Christiane Szekat, Gabriele Bierbaum, Christoph Bourauel

**Affiliations:** 1 Oral Technology, Medical Faculty, University Hospital Bonn, Bonn, Germany; 2 Department of Orthodontics, Medical Faculty, University Hospital Bonn, Bonn, Germany; 3 Institute of Medical Microbiology, Immunology, and Parasitology, Medical Faculty, University Hospital Bonn, 53127 Bonn, North Rhine-Westphalia Germany

**Keywords:** Irreversible hydrocolloids, *Boswellia sacra*, Green synthesis, Antimicrobial activity, Nanotechnology

## Abstract

**Objectives:**

Disinfection of alginate impression materials is a mandatory step to prevent cross-infection in dental clinics. However, alginate disinfection methods are time-consuming and exert a negative impact on accuracy and mechanical properties. Thus, this study aimed to prepare disinfecting agents (CHX and AgNO_3_) and silver nanoparticles reduced by a natural plant extract to produce a self-disinfecting dental alginate.

**Methods:**

Conventional alginate impression material was used in this study. Silver nitrate (0.2% AgNO_3_ group) and chlorohexidine (0.2% CHX group) solutions were prepared using distilled water, and these solutions were later employed for alginate preparation. Moreover, a 90% aqueous plant extract was prepared from *Boswellia sacra* (*BS*) oleoresin and used to reduce silver nitrate to form silver nanoparticles that were incorporated in the dental alginate preparation (*BS*+AgNPs group). The plant extract was characterized by gas chromatography/mass spectrometry (GC/MS) analysis while green-synthesized silver nanoparticles (AgNPs) were characterized by UV-visible (UV–vis) spectroscopy and scanning electron microscopy (SEM). An agar disc diffusion assay was used to test the antimicrobial activity against *Candida albicans, Streptococcus mutans*, *Escherichia coli,* methicillin-resistant and susceptible *Staphylococcus aureus* strains, and *Micrococcus luteus.* Agar plates were incubated at 37 ± 1 °C for 24 h to allow microbial growth. Diameters of the circular inhibition zones formed around each specimen were measured digitally by using ImageJ software.

**Results:**

Chemical analysis of the plant extract revealed the presence of 41 volatile and semi-volatile active compounds. UV–Vis spectrophotometry, SEM, and EDX confirmed the formation of spherical silver nanoparticles using the *BS* extract. CHX, AgNO_3_, and the *BS*+AgNPs modified groups showed significantly larger inhibition zones than the control group against all tested strains. *BS*+AgNPs and CHX groups showed comparable efficacy against all tested strains except for *Staphylococcus aureus*, where the CHX-modified alginate had a significantly higher effect.

**Conclusions and clinical relevance:**

CHX, silver nitrate, and biosynthesized silver nanoparticles could be promising inexpensive potential candidates for the preparation of a self-disinfecting alginate impression material without affecting its performance. Green synthesis of metal nanoparticles using *Boswellia sacra* extract could be a very safe, efficient, and nontoxic way with the additional advantage of a synergistic action between metal ions and the phytotherapeutic agents of the plant extract.

## Introduction

Many materials are available nowadays to take primary and secondary impressions for patients in dental clinics [1]. The choice of an impression material for a particular situation is mainly dependent on the treatment protocol and the operator’s preference. Meanwhile, hydrocolloids and elastomeric polymers are the most used impression materials for various dental treatments [2]. Hydrocolloid dental materials include both agar–agar and alginates, which are viscous liquids, present in a sol state or a gelatinous consistency.

Alginates are salts of alginic acid, a polysaccharide extracted from the cell walls of brown algae. They belong to the Phaeophyceae family, which is widespread, especially in the colder oceans of the Northern Hemisphere [3]. Dental alginates are irreversible elastic hydrocolloids that were developed in the 1940s when the agar impression material was limited [4]. Irreversible alginates consist of salts of alginic acid, calcium sulfate as a reactor, zinc oxide, potassium titanium fluoride, diatomaceous earth, and coloring or flavoring agents [5]. Alginate is provided in the form of a powder to be mixed with water and set by a chemical reaction that cross-links the carbohydrate polymer, forming a hydrogel [5].

Alginates are one of the most frequently and routinely used dental materials in every dental practice, especially at the first dental visit for a pre-treatment evaluation. Their common usage is due to their cost-effectiveness, ease of use, and fast setting time that can be even controlled by temperature [6]. Disadvantages include less accurate reproduction of details as compared with elastomeric impression materials, poor dimensional stability, and inadequate retention of non-perforated trays [7]. Moreover, hydrocolloids are hydrophilic by nature; therefore, they swell if immersed in water or disinfectant; and thus; their dimensional stability upon disinfection and storage is problematic [8].

Dental impressions present a source of cross-infection to the dentist and dental technicians since they are exposed to blood and saliva inside the oral cavity; hence, disinfection is mandatory for impression materials [9]. On the other hand, distortion can be a problem if disinfection guidelines are not strictly followed. Disinfectant sprays are used for alginate impressions, but they do produce air bubbles in the cast, thereby affecting accuracy [10]. Although immersion in disinfectants like 1% sodium hypochlorite or 2% glutaraldehyde can result in dimensional changes of only 0.1%, still the quality of the impression surface may be greatly compromised if the recommended period and other factors are not strictly controlled [11, 12].

Attempts to incorporate disinfectants into the alginate powder or mixing water were found to be an effective way of disinfection with minimal adverse effects on dimensional accuracy and surface details [13, 14]. Follow-up studies of irreversible hydrocolloid impression materials pre-impregnated with disinfectants have shown that this technique saves time, is active against oral pathogens, and demonstrates greater dimensional stability than spray and immersion techniques [15].

The antibacterial activity of metal and metal oxide nanoparticles is extensively studied in medicine [16, 17]. Silver and its compounds have been used as antimicrobial agents for various medical purposes. Silver ions are effective against bacteria, viruses, and fungi besides causing no harm to humans at low concentrations [18]. Materials with at least one external dimension of 1–100 nm are defined as nanomaterials or nanoparticles (NPs), and they have attracted increasing interest in recent years, especially in dentistry [19]. Silver nanoparticles have demonstrated unique and significantly different physical and chemical properties compared to their macroscopic counterparts. The smaller the nanoparticles, the greater the surface-to-volume ratio, dispersion, and antimicrobial efficacy [19].

Conventional approaches for the production of nanoparticles (NPs) are typically expensive, toxic, complicated, and non-ecological [20]. Green nanotechnology is a recent approach, which utilizes microorganisms, plants, or their extracts as reducing and capping agents in the synthesis of AgNPs. Plants play an important role in the biosynthesis of NPs and their major advantage is that they are easily available, safe, and contain a variety of metabolites that can contribute to the reduction of silver ions [20]. Green nanoparticles exhibit distinct characteristics compared to those generated through physical and chemical means. The green process employs a bottom-up approach to create magnetic nanoparticles using biological constituents like plant extracts or bacteria to replace the costly chemical-reducing agents. The eco-friendly transformation of microparticles into NPs through green reduction is environmentally favorable, sustainable, devoid of chemicals, cost-effective, and scalable. Additionally, green synthesis leads to the recovery and recycling of valuable metal salts like gold (Au) and silver (Ag) from waste streams [21, 22]. *Boswellia sacra (B. sacra)* is a tree in the genus *Boswellia* from which frankincense oleo gum resin is collected. It is native to Oman, Yemen, and Somalia. *B. sacra* finest sorts are presented under the local names Houjri, Najdi, and Sahli or Shaebi, based on the region of cultivation in Oman. Houjri is the first-grade, most expensive resin that is growing in the north of the Samhan Mountains [23]. Resin essential oils contain several pharmacologically active compounds (monoterpenes, sesquiterpenes monoterpenes, sequiterpinols, and ketones) that have antimicrobial activity against important human pathogens, both bacterial and fungal organisms, such as *Staphylococcus aureus*, *Escherichia coli*, *Proteus vulgaris*, and *Candida albicans* [23].

To the best of our knowledge, *Boswellia sacra (B. sacra)* plant extract has never been used for the green synthesis of silver nanoparticles and has not been incorporated before or used for the disinfection of any hydrocolloid impression material in dentistry. Therefore, in our previous and present investigations, we aimed to replace the water used for the preparation of alginate with a prepared aqueous solution of *B. sacra* added to silver nitrate in a given concentration to biosynthesize nanoparticles for enhanced antimicrobial activity. Moreover, a 0.2% silver nitrate solution and a 0.2% CHX solution were used for the preparation of two other antimicrobial-modified groups for comparison.

Our former results which assessed our modification on alginate physical and mechanical properties showed that detail reproduction and accuracy of alginate were not negatively impacted by the different self-disinfection modifications. Moreover, elastic recovery was improved by the addition of CHX, AgNO_3_, and *BS* + AgNPs. Additionally, it was found that all groups reported tear strength values that were within the acceptable range, with CHX and *BS* + AgNPs showing significantly higher tear strength values compared to the control group [24].

Therefore, based on the fact that the green synthesis of metal nanoparticles using the *Boswellia sacra* extract did not alter the functional performance of alginate, this study aimed to chemically characterize *B. sacra* extract and to confirm the production of the green-synthesized nanoparticles using color change, UV–Vis spectroscopy, and scanning electron microscopy (SEM). Moreover, the effect of the modifications was tested along with control alginate against six microbial strains to confirm its efficacy.

## Materials and methods

### Materials

 Materials and microbial strains used in the present study are listed in Table 1.

**Table 1:** Materials used in the study

Material	Supplier information
Conventional fast set alginate	Pluradent GmbH and Co., Bornheim, Germany.
Superior Hojari frankincense*, Boswellia sacra* gum	Dohfar mountains, Oman (imported by Jeomra Verlag, Georg Huber, Hessen, Germany).
Silver nitrate ≥ 99.0%	Sigma-Aldrich, St. Louis, MO, USA 209139-25G
Chlorhexidine digluconate powder	Caymen Chemical, Biomol GmbH, Hamburg, Germany.
Microbial strains	Strain number
1. *Streptococcus mutans* 2. *Staphylococcus aureus* 3. *Escherichia coli* 4. *Candida albicans* 5. *Micrococcus luteus* 6. *Staphylococcus aureus*	DSM 20523 USA 300 NRS384 (methicillin-resistant) BW 25113 DSM 70014 ATCC 4698 SG511 (methicillin-susceptible)

### Methods

#### Preparation of the silver nanoparticles in *Boswellia sacra (B. sacra)* extract

Frankincense resin (50 g) was washed, dried, and frozen overnight. The freezing step enabled the grinding of the resin in a blender to a fine powder. Afterward, the resin powder was soaked in an ethanol/water mixture (90% water : 10% ethanol) for 3 days and extracted using a Soxhlet extractor for 7 h. Filtration of the extract was performed using Whatman’s paper 1. Afterward, the extract was stored at 4 °C until usage.

**Fig. 1:**
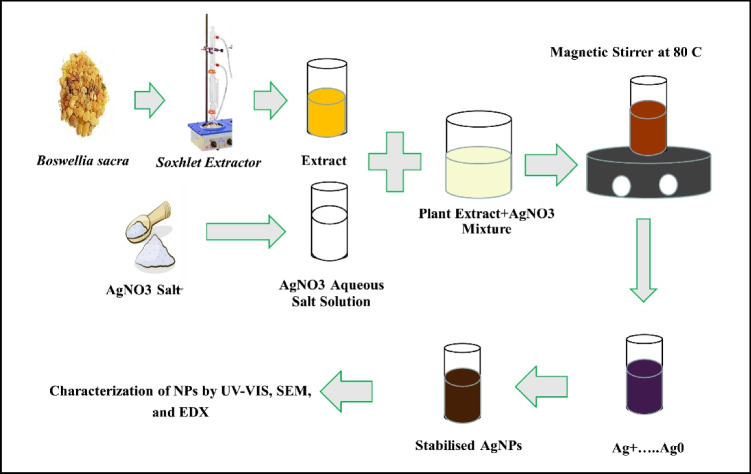
Schematic diagram showing steps of preparation of *B. sacra* extract and green nanoparticles

For preparation and reduction of the silver nanoparticles, 10 ml of the filtrate was added to 30 ml of 0.2% AgNO_3_ solution in a conical flask with continuous stirring at a speed of 400 rpm. The mixture was incubated for 3 days at room temperature in the darkness, with the solution color turning from white to brown, demonstrating the formation of AgNPs (Fig. 1).

#### Preparation of 0.2% silver nitrate and 0.2% CHX solutions

0.2% AgNO_3_ solution was prepared by adding 2 g of 99% silver nitrate powder to 250 ml of distilled water and mixing on a vortex mixer for a few minutes. Afterward, 750 ml of distilled water was added to the mixture and vortexed for another few minutes. For the 0.2% CHX solution, 2 g of chlorohexidine powder was added to 1000 ml distilled water and vortexed for 15 min for complete dissolution of the powder in the liquid.

#### Specimen’s preparation and grouping

A sensitive balance was used to weigh the alginate powder for each specimen to be mixed with a specific amount of the designated liquid to prepare four different groups (water (control), 0.2 % AgNo_3_, 0.2 % CHX, and *BS*+ AgNPs) for testing and comparison. Hand mixing was carried out at 23 ± 1 °C, and relative humidity until a homogenous mixture was obtained according to the manufacturers’ instructions.

#### Chemical analysis of plant extract mixture (GC/MS)

One milliliter of *BS* extract was injected to analyze its chemical constituents at the Agriculture Research Center, Giza, Egypt using a gas chromatograph (GC) (Agilent Technologies 7890A) attached with a mass spectrometer (MS) (MSD, Agilent 7000). The GC was equipped with a polar Agilent HP-5%-phenyl methyl poly siloxane and a capillary column of 0.25-mm inner diameter, and 0.25-μm film thickness. The injector temperature was set at 200 °C while the detector temperature was set at 250 °C. Helium was the carrier gas with a linear velocity of 1 ml/min. Mass spectra had an acquisition mass range of 50–800 *m*/*z* and an interface temperature of 250 °C. The quantification of components was carried out using a percent relative peak area. Active compounds were identified by comparing their relative retention times with authentic compounds and by computer matching with the NIST and WILEY library as well as by relating the spectral data with those described in the literature [25].

#### Characterization of the Ag nanoparticles

##### Color change and ultraviolet-visible (UV–Vis) spectroscopy

Bioreduction of the prepared mixture was observed by monitoring the color changes from white to dark brown visually and by using UV–visible spectrophotometer (UviLine 9400, Schott, Mainz, Germany). Spectroscopy absorption analysis was performed by measuring 1 ml aliquots at different time intervals. Samples were scanned in the 200–700 nm range, with a scanning speed of 475 nm/min, and at 1-cm optical path at room temperature. UV–vis absorption spectra measurements started 1 h after incubating the AgNO_3_ solution with *B. sacra* extract and distilled water was used as a blank reference [26, 27].

#### Scanning electron microscopy (SEM)

The structures, sizes, and shapes of the nanoparticles were examined using a scanning electron microscope (SEM, Philips XL 30, Philips, Eindhoven, The Netherlands). A thin film of the prepared *BS*+SNPs solution was placed on a carbon-coated copper grid and allowed to dry in the air for 1 h. The SEM operated at 10 kV and magnifications of 1200×, and 2000×, using spot size 3. The measurements of the different spherical particles and clusters were taken using Image J software (Wayne Rasband (NIH), Version 1.53k) [28].

#### Antimicrobial activity analysis

Agar well diffusion assays were used to assess the antimicrobial activity against *Streptococcus mutans* (*S. mutans*), methicillin-resistant *Staphylococcus aureus* (MRSA*), Escherichia coli (E. coli), Candida albicans (C. albicans)* and *Micrococcus luteus (M. luteus)*, and *Staphylococcus aureus (S. aureus).* Müller–Hinton agar plates were overlaid with 0.1 ml of suspensions of the indicator strains that had been adjusted to an OD_600_ of 0.1 using a UV–Vis spectrophotometer (UviLine 9400). After 10 min, the suspensions were removed by pipetting and the plates were left to dry for 20 min.

For each modification, three replicates of freshly mixed alginate were prepared according to the manufacturer’s instructions using sterile plastic spatulas. Wells (5-mm diameter) were punched into each agar plate using a sterile cork borer to receive the freshly mixed, unset different alginate samples. The antimicrobial activities of the tested samples were assessed after incubating the plates (Heraeus GmbH & Co. KG, Hanau, Germany) at 37 ± 1 °C for 24 h [29]. The diameters of the circular inhibition zones formed around each specimen were measured digitally. High-resolution digital pictures of all Petri dishes were taken using a Canon T3i (Canon Corp, NY, USA) digital single-lens reflex camera with an 18–55 mm lens, which allowed a magnification of up to 35×. The camera was fixed on a tripod to standardize the shooting angle and to standardize the distance between the camera lens and plates. Additionally, a base was prepared with reference points to ensure standardization of the plates’ placement. High-resolution digital pictures were transferred to a computer, and ImageJ (Wayne Rasband (NIH), Version 1.53k) software was used to measure inhibition zones. Pictures were rescaled and the value of each inhibition zone was taken three times [30].

#### Statistical analysis

Results are presented as mean and standard deviation (SD). The Shapiro–Wilk normality test was used to examine whether the variables follow a normal distribution. All quantitative variables showed parametric distribution; therefore, one-way analysis of variance (ANOVA) was used for comparison between the groups. Tukey’s post hoc test was used employed for pairwise comparison between the groups when the ANOVA test was significant. The significance level was set at *p* ≤ 0.05. Statistical analysis was performed using Minitab 17.1.0 for Microsoft Windows.

## Results

### Chemical analysis

Gas chromatography/mass spectrometry revealed the presence of 41 volatile and semi-volatile active compounds (Table 2).

**Table 2:** Results of GC/MS analysis of *Boswellia sacra* extract

	Retention time (min)	Compounds	Area (%)
1	5.166	Thuja-2,4(10)-diene	1.09
2	6.811	Myrtenol	1.54
3	6.913	L-Pinocarveol	2.86
4	6.95	cis-p-Mentha-2,8-dien-1-ol	2.58
5	7.016	Carveol	1.34
6	7.471	α-Thujenal	0.98
7	7.626	p-Cymen-7-ol	2.34
8	8.262	Isobornyl acetate	1.19
9	9.222	Germacrene D-4-ol	0.61
10	9.271	β-Elemen	0.82
11	10.075	α-Selinene	1.58
12	10.681	Epiglobulol	0.51
13	10.837	Caryophyllene oxide	1.08
14	11.411	Longifolene	2.17
15	12.424	Lanceol, cis	0.36
16	13.736	Farnesol	0.7
17	13.868	Cembrene	0.27
18	14.343	Geranyl isovalerate	0.55
19	14.737	Ledol	1.49
20	14.938	Heptacosane	1.58
21	15.615	Hexacosane	3.67
22	16.345	Vitexin	0.35
23	16.418	Tetracosane	6.13
24	16.997	Quinidine	0.33
25	17.399	Phytan	9.87
26	17.78	β-Santalol	0.73
27	18.079	Chamigrene	1.5
28	18.436	cis-Sesquisabinene hydrate	0.61
29	18.625	Crocetane	12.55
30	19.441	Betulin	0.7
31	19.527	Phytol	1.16
32	20.126	Hexadecane	13.38
33	20.884	Tetradecane, 2,6,10-trimethyl-	0.59
34	21.02	Nonacosane	0.52
35	21.295	Octacosane	10.62
36	21.975	Hexa-hydro-farnesol	0.87
37	22.102	Squalane	0.96
38	22.398	Pentadecane	7.95
39	22. 456	Boswellic acid	0.99
40	22.845	Stigmasterol	0.99
41	23.177	Eicosanoic acid	0.88

### Characterization of the Ag nanoparticles

#### Color change and UV-visible spectroscopy analysis

**Fig. 2:**
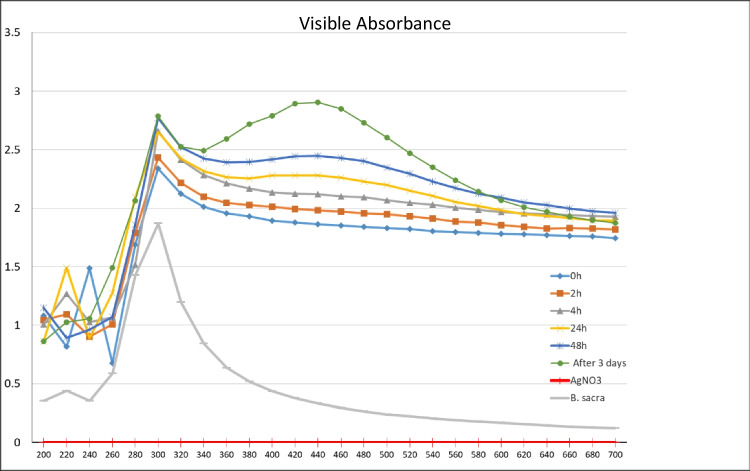
UV–Vis absorption spectra of Ag NPs green synthesized by *Boswellia sacra* extract at different time intervals

The biological synthesis of AgNPs using *B. sacra* extract as a reducing and stabilizing agent was confirmed primarily by the color change from white to light brown and then dark brown. Silver nanoparticle surface plasmon excitation causes the color change in the solution, which is the key and notable evidence for the formation of Ag NPs. Several UV–Vis absorption spectra were measured after 0, 2, 4, 24, and 48 h and 3-day intervals (Fig. 2). An absorption peak localized at 300 nm was observed which is supposed to be related to *B. sacra* extract. Furthermore, the absorption spectra showed a maximum absorbance peak at 440 nm which is related to the localized surface plasmon resonance of AgNPs. A gradual increase in the intensity (2.119, 2.281, 2.449, and 2.906 a.u) as a function of time was also observed, indicating an increase of the biosynthesized AgNPs with the maximum intensity being observed after 3 days.

#### Scanning electron microscopy

**Fig 3:**
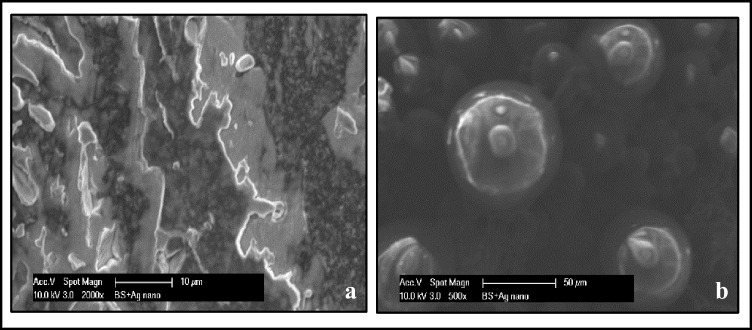
SEM magnified images: **a** AgNO_3_ and (**b**) *Boswellia sacra* extract

**Fig. 4:**
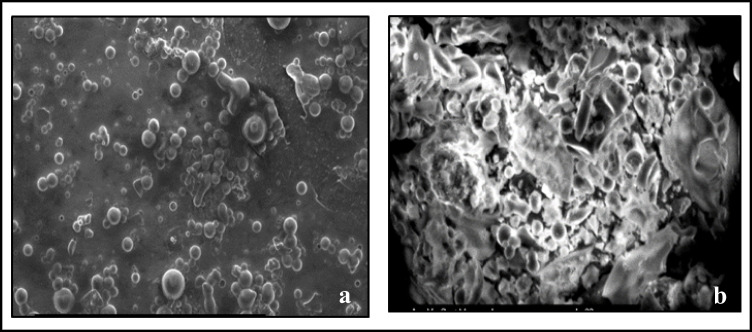
**a** SEM magnified image (500x) confirming the formation of spherical nanoparticles and nanoclusters in-between micro particles. **b** SEM magnified image (1500x) showing clusters and different sizes of silver nanoparticles sizes, microparticles, and *B. sacra* constituents

The morphological images of Ag NPs produced by green synthesis are shown in Figs. 3 and 4. The results indicate the presence of spherical nanoparticles, as well as clusters of spherical nanoparticles with different diameters ranging from 50 to 100 nm.

### Antimicrobial activity

**Fig. 5:**
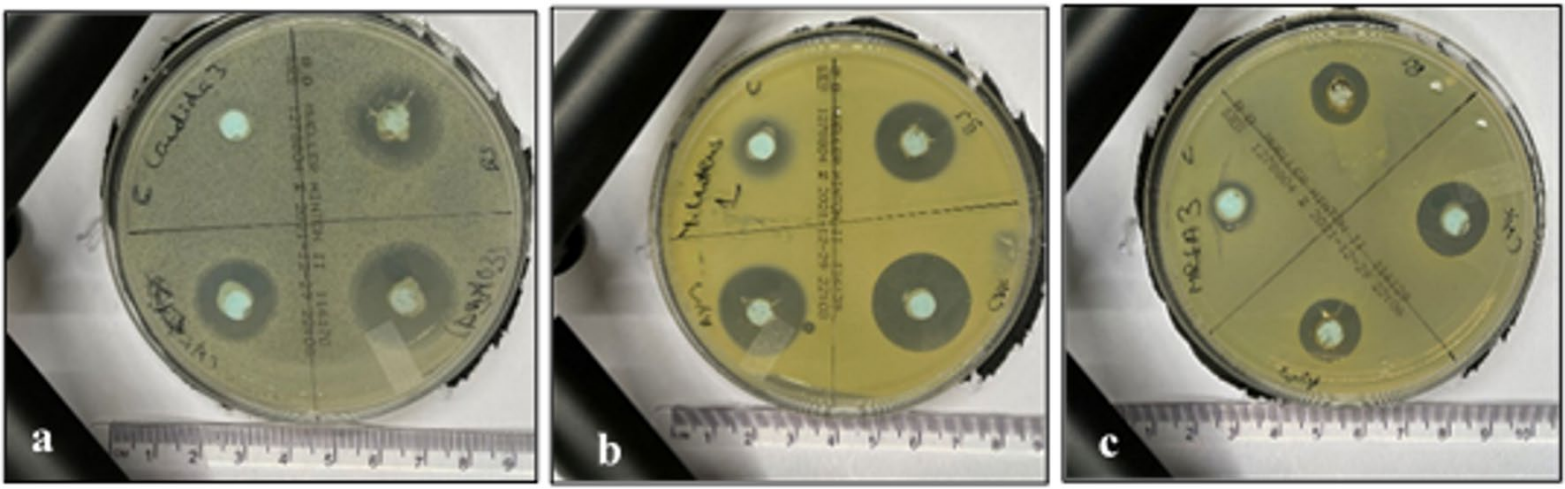
Inhibition zones for all four tested groups against **a** *C. albicans*, **b** *M. luteus*, and (**c**) the MRSA *S. aureus* USA300

**Fig 6:**
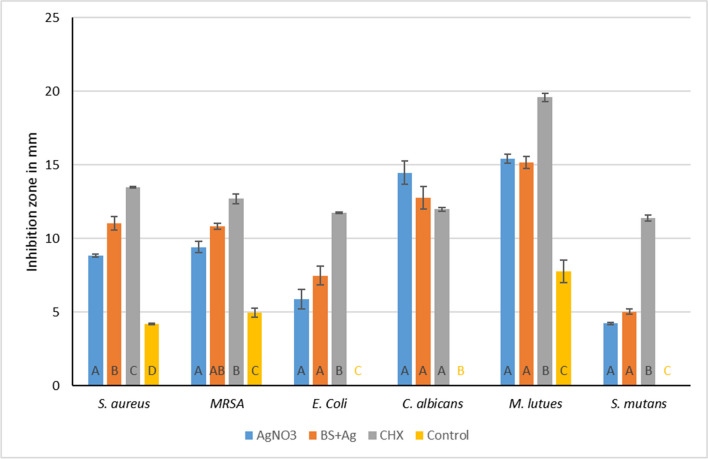
Bar chart representing the mean inhibition zone values and standard of error of the four tested alginate groups

Agar diffusion tests were performed in triplicates. The variables showed a parametric distribution, and thus, one-way ANOVA was used to test the antimicrobial effect of *BS+Ag* (AgNPs*)*, CHX, AgNO_3_, and control groups against the six different microbial strains followed by Tukey’s post hoc test for pairwise comparison between the tested groups (Figs. 5 and 6). The ANOVA indicated that there was a statistically significant antibacterial effect of the three modified groups’ alginates against all strains, *F*(4, 30) = 63.23, *p*-value < 0001. Post hoc comparison using Tukey’s test indicated that the three modified groups’ mean values showed significantly higher antibacterial activities than the control (Fig. 5).

Mean values (Fig. 6) against these strains were as follows: *S. aureus* (control = 4.2 mm, SD ± 0.2; *BS+*AgNPs = 11.0 mm, SD ± 1.3; CHX*=* 13.5 mm, SD ± 0.1; AgNO_3_ = 8.8 mm, SD ± 0.3), *M. luteus* (control = 7.8 mm, SD ± 2.2; *BS*+AgNPs = 15.2 mm, SD ± 1.2; CHX *=* 19.6 mm, SD ± 0.8; AgNO_3_ = 15.4 mm, SD ± 0.9), whereas *S. mutans* (control = 0.0 mm, SD ± 0.0; *BS+*AgNPs = 5.0 mm, SD ± 0.5; CHX = 11.4 mm, SD ± 0.6; AgNO_3_ = 4.3 mm, SD ± 0.2) and MRSA (control = 5.0 mm, SD ± 0.9; *BS+*AgNPs = 10.9 mm, SD ± 0.6; CHX = 12.7 mm, SD ± 1; AgNO_3_ = 9.4 mm, SD ± 1.2). Furthermore, against *E. coli*, there was a statistically insignificant difference between the mean values of *BS+*AgNP (*M* = 7.5 mm, SD ± 1.9) and AgNO_3_ (*M* = 5.9 mm, SD ± 2), whereas, CHX (*M* = 11.8 mm, SD ± 0.2) showed the highest mean values. No significant differences were observed between the three modified groups against *C. albicans* (control = 0.0 mm, SD ± 0.0; *BS+*AgNPs = 12.8 mm, SD ± 2.2; CHX = 12.0 mm, SD ± 0.4; AgNO_3_ = 14.5 mm, SD ± 2.4).

## Discussion

Alginate impressions are regularly contaminated with patients’ blood and saliva, thus acting as a potential medium for the transfer of infectious microorganisms and viruses between patients, operators, and dental auxiliaries [9]. The presence of numerous microbes, including streptococci*,* staphylococci*, Candida*, *Pseudomonas aeruginosa*, and MRSA on alginate impressions and gypsum casts has been documented and can be a cause of infections in dental clinics [31]. Since alginates undergo syneresis and imbibition according to the surrounding conditions, post-setting disinfection by spraying or immersion often compromises the accuracy of alginate impressions [32].

The difficulties associated with disinfecting irreversible hydrocolloid impression materials have directed this study to develop a self-disinfecting dental alginate material by mixing alginate powder with silver nanoparticles synthesized by *Boswellia sacra* extract. On the other hand, dental alginate was modified with 0.2% CHX and 0.2% silver nitrate solutions as well for self-disinfection. The three modified groups were tested and compared with unmodified dental alginate as a control.

In the present study, a color change was observed over time after mixing *BS* extract and silver nitrate solution. The color change resulted from the excitation of surface plasmon resonance (collective movement of free electrons in the silver when light falls on it) due to the reduction of Ag^2+^ ions to Ag 0 by biomolecules such as phenols, flavonoids, ketones, tannins, and proteins present in *BS* extract [33]. These phytochemicals contain hydroxyl and ketone groups that induce the reduction of Ag ions to form appropriate nuclei that grow during the development phase into spherical AgNPs [34].

Furthermore, UV–Vis spectrophotometry confirmed the formation of AgNPs by yielding a bell-shaped spectrum after the different time intervals. The broad plasmon band may be due to the presence of plant metabolites in the solution, which may adsorb the light in this spectrophotometric range as well. The peak was observed at 440 nm, and an absorbance between 400 and 460 nm is always characteristic for the formation of silver nanoparticles [35]. It is worth mentioning that spherical nanoparticles show only a single SPR band and the number of peaks increases with an increasing range of particle shapes [36, 37]. SEM magnified images of samples have shown that the particles were mostly spherical with a size distribution in the range of 50 to 100 nm. Agglomerated AgNPs were also present, which may be a sign of sedimentation [38].

The antimicrobial activity of the modified groups was tested using agar diffusion assays against different microbial strains including four Gram-positive bacteria (*S. aureus,* methicillin-sensitive and resistant, *S. mutans*, and *M. luteus*), one Gram-negative bacterium (*E. coli*) and a yeast (*C. albicans*)*.* The three modified groups were significantly more active than the unmodified alginate against all microbial strains. Results showed that the *BS*+AgNPs group differed insignificantly from AgNO_3_ only against all strains except *S. aureus.* On the other hand, the antimicrobial activity of *BS*+AgNPs was comparable to the CHX group against *C. albicans* and MRSA. CHX showed significantly higher activity than all other groups against *S. mutans*, *S. aureus*, *E. coli*, and *M. luteus.* The unmodified alginate also showed a reproducible weak antibacterial activity against both *S. aureus* strains and *M. luteus*, which might be due to the presence of zinc ions in the alginate powder.

The results are in agreement with several studies, which reported that the incorporation of disinfecting agents such as silver nanoparticles, quaternary ammonium compounds, chlorhexidine, iodine, bisguanidine compounds, and ammonium chloride into the impression materials eliminates the need for separate disinfection of the impression after removal from the mouth [39, 40]. Our results showed also that *BS*+AgNPs were efficient against an MRSA which is an antibiotic-resistant strain and thus might have the potential to be used in medicine. Profound antimicrobial activities of green-synthesized AgNPs were previously reported by Vanlalveni et al. [41], Akhtar et al [42], and Tahmasebi et al. [43].

The antimicrobial activity of *BS*+AgNPs could be due to the synergistic action of both the plant extract and AgNPs. Different phytochemical constituents were identified by gas chromatography/mass spectrometry in this and a previous study [44]. Terpenoids (e.g., boswellic acids, cymene-7-ol, L-pinocarveol, carveol, and cis-sesquisabinene hydrate) were detected in considerable amounts in the *B. sacra* extract. Although the antibacterial mode of action of terpenes remains mostly unknown, it has been reported that most terpenoids act by partitioning into the membrane, increasing its permeability and dissipating proton motive force [45].

Carveol, for example, has been shown to affect the membrane integrity in *E. coli* and *S. aureus* and induce leakage of potassium from *S. aureus* cells [46]. Moreover, alkaloids (e.g., quinidine), sterols (e.g., stigmasterol), flavonoids (e.g., vitexin), phenols (e.g., cis-p-Mentha-2, 8-dien-1-ol), and saponins (e.g., squalane) were detected. Many of these secondary plant compounds have long-established antimicrobial activities, e.g., saponins damage the bacterial cell membrane [47], stigmasterol is bacteriostatic for MRSA [48], and vitexin inhibits biofilm formation of *P*. *aeruginosa* and alters the surface properties of *S. aureus*) [49].

Silver in a nanometre scale of less than 100 nm is toxic to a wide range of microorganisms [50]. Although the exact mechanism of silver nanoparticles’ antibacterial effects has not been completely clarified, various antibacterial actions have been proposed [51]. Silver nanoparticles can penetrate bacterial cell walls, damage the cytoplasmic membrane, and even result in cell lysis [50]. There is also an influence of the particle size and shape on the release of silver ions since AgNPs with spherical or quasi-spherical format are more susceptible to silver release, due to their larger surface area [51]. Moreover, Gram-negative bacteria are more susceptible to silver nanoparticles than Gram-positive strains. The cell walls of Gram-positive bacteria are composed of a thick peptidoglycan layer of linear polysaccharide chains cross-linked by short peptides, thus forming a more rigid structure leading to difficult penetration of the AgNPs compared to the Gram-negative bacteria, where the cell wall possesses thinner peptidoglycan layers [52, 53].

Silver nitrate, which is a suspension of sub-microscopic silver ions, can significantly reduce the duration and severity of many bacterial infections [54]. Silver is an inert metal but it is biologically active in an aqueous environment in which it is present in an ionic soluble state (Ag^+^) [55]. One of the main advantages of silver is its oligo dynamic effect, of having high microbicidal capacity in water at a very low concentration (one part per million) [55]. One of the important mechanisms of Ag^+^ toxicity is the ability of silver ions to interact with the bacterial inner membrane and impair its integrity. Additionally, silver ions target the SH groups on proteins, disrupt their disulfide bonds, and inactivate dehydratases by breaking down 4Fe-4S clusters [56]. A recent study showed that in *S. aureus*, silver ions target proteins involved in glycolysis, pentose phosphate cycle, and defense against ROS [57].

The toxicity of silver nitrate is dosage-dependent; oral ingestion of more than 2 g of silver nitrate can be fatal [54]. Silver nitrate rapidly reacts with chloride yielding extremely non-soluble silver chloride that causes a fatal electrolyte imbalance. However, the dosage of silver nitrate used in this study (0.2%) is very low, and for the *BS*+AgNPs group, it was even lower. One milliliter of 0.2% silver nitrate solution contains 0.002 g silver nitrate. A 38-ml solution that is used to make a full arch impression contains 0.08 mg silver nitrate and is equivalent to 0.33% of a fatal dose. In addition, it should be taken into consideration that silver nitrate was not used in a free form or ingested, but instead, it went through a chemical reaction and is enclosed in a gel that is used topically inside the mouth and not in a liquid form.

Chlorhexidine in the present study was used as it has a proven antimicrobial activity against various microbial strains [58]. The selected concentration of CHX (0.2%) is believed to be effective for oral disinfection and plaque inhibition with no serious side effects [58, 59]. The positively charged chlorhexidine works actively against bacteria by binding to the negatively charged sites on the bacterial cell wall causing destabilization of the cytoplasmic membrane and may affect membrane proteins [60, 61]. The bacterial uptake of chlorhexidine is very fast, typically functioning within 20 s. For *C. albicans* (fungus), the mechanism of action is almost the same in that the fungus uptakes chlorhexidine rapidly and damages the integrity of the cell wall and the plasma membrane resulting in leakage and cell death [62].

It is important to note that impression materials that come into contact with contaminated saliva and blood can pose a substantial risk of cross-contamination, not only with bacteria and fungi but also with highly contagious viruses like hepatitis B, hepatitis C, herpes, and HIV. As a result, there are plans to conduct further research to assess the effectiveness of the green-modified alginate against these viruses.

## Conclusions

Within the context of this study, it can be concluded that the usage of *Boswellia sacra* plant extract was effective for the biosynthesis of AgNPs in a simple, inexpensive, and ecologically friendly way at room temperature. The analysis of the *BS* extract confirmed the presence of different organic compounds that can act as reducing and stabilizing agents for the biosynthesis of AgNPs. Furthermore, the antimicrobial activity of dental alginate was enhanced by the incorporation of 0.2% CHX, 0.2% AgNO_3_, and green-synthesized AgNPs against all tested microbial strains. Future work involving testing the effect of the three used antimicrobial agents on the dimensional accuracy, detail reproduction, tear strength, elastic recovery, setting time, and flow of alginate is in progress.

## Data Availability

The datasets used and/or analyzed during the current study are available from the corresponding author upon reasonable request.
